# Potential Suppressive Effect of Nicotine on the Inflammatory Response in Oral Epithelial Cells: An In Vitro Study

**DOI:** 10.3390/ijerph18020483

**Published:** 2021-01-09

**Authors:** Na An, Jasmin Holl, Xuekui Wang, Marco Aoqi Rausch, Oleh Andrukhov, Xiaohui Rausch-Fan

**Affiliations:** 1 Department of General Dentistry II, Peking University School and Hospital of Stomatology, Beijing 100081, China; anna@pkuss.bjmu.edu.cn (N.A.); 1711210589@pku.edu.cn (X.W.); 2 Competence Center for Periodontal Research, University Clinic of Dentistry, Medical University of Vienna, 1090 Vienna, Austria; jasminholl@me.com (J.H.); marco.rausch@meduniwien.ac.at (M.A.R.); xiaohui.rausch-fan@meduniwien.ac.at (X.R.-F.); 3 Department of Periodontology, Peking University School and Hospital of Stomatology, Beijing 100081, China

**Keywords:** nicotine, epithelial cell, inflammation, lipopolysaccharide

## Abstract

Smoking is a well-recognized risk factor for oral mucosal and periodontal diseases. Nicotine is an important component of cigarette smoke. This study aims to investigate the impact of nicotine on the viability and inflammatory mediator production of an oral epithelial cell line in the presence of various inflammatory stimuli. Oral epithelial HSC-2 cells were challenged with nicotine (10^−8^–10^−2^ M) for 24 h in the presence or absence of *Porphyromonas gingivalis* lipopolysaccharide (LPS, 1 µg/mL) or tumor necrosis factor (TNF)-α (10^−7^ M) for 24 h. The cell proliferation/viability was determined by MTT assay. Gene expression of interleukin (IL)-8, intercellular adhesion molecule (ICAM)-1, and β-defensin was assayed by qPCR. The production of IL-8 protein and cell surface expression of ICAM-1 was assessed by ELISA and flow cytometry, respectively. Proliferation/viability of HSC-2 cells was unaffected by nicotine at concentrations up to 10^−3^ M and inhibited at 10^−2^ M. Nicotine had no significant effect on the basal expression of IL-8, ICAM-1, and β-defensin. At the same time, it significantly diminished *P. gingivalis* LPS or the TNF-α-induced expression levels of these factors. Within the limitations of this study, the first evidence was provided in vitro that nicotine probably exerts a suppressive effect on the production of inflammatory mediators and antimicrobial peptides in human oral epithelial cells.

## 1. Introduction

Periodontal disease is a complex multifactorial disorder characterized by oral dysbiosis and dysregulated host immune response, the combined effects of which lead to the destruction of tooth-supporting tissue [1,2,3]. *Porphyromonas gingivalis* (*P. gingivalis*) is a gram-negative anaerobic bacterium that has been considered as a keystone pathogen in periodontitis development and progression [4]. The oral epithelium is the primary physical barrier to infections and is in continuous contact with potentially pathogenic bacterial biofilms in vivo. *P. gingivalis* is known to influence the structural and functional integrity of gingival epithelium, which results in the disruption of homeostasis and contributes to the progression of periodontal disease [5,6,7]. Besides barrier function, the oral epithelium produces several factors that are involved in the maintenance of oral health and antibacterial defense. Particularly, the production of interleukin (IL)-8 and intercellular adhesion molecule 1 (ICAM)-1 is involved in the process of leukocyte infiltration and transepithelial migration [8,9]. Production of antimicrobial peptides by epithelium plays an essential role in regulating bacterial growth [10].

Smoking is a widely accepted risk factor for periodontal disease, influencing its incidence and severity [11,12]. Nicotine is considered the most pharmacologically active compound in tobacco smoke and is primarily involved in developing smoking habits. Nicotine’s specific effects are hard to disentangle from the effects of the many other harmful components in cigarette smoke [13]. Understanding the nicotine effect in the oral cavity is also of great importance because of the increasing availability of nicotine replacement therapy and the popularity of novel nicotine-containing products such as e-cigarettes [14].

The oral epithelium is the first tissue exposed to nicotine following tobacco smoking. Several pathways have been implicated in the deleterious effect of nicotine on periodontal cells homeostasis, including increasing autophagy and apoptosis in periodontal ligament cells via nicotinic acetylcholine receptor α7 and β4 subunits [15,16], modifying the migration of human gingival epithelial cells through the activation of the MAPK ERK1/2 and p38 signaling pathways [17], inducing the production of cyclooxygenase-2 and prostaglandin E2 in osteoblasts via the JAK/STAT pathway [18]. Until now, the effect of nicotine on the oral epithelium remains conflicting [19,20], with some findings reporting no influence on inflammatory factor production [21].

The secretory function of epithelium is substantially affected by various inflammatory stimuli, particularly bacterial components or inflammatory cytokines. Such a situation is particularly characteristic of periodontal disease. The effect of nicotine on oral epithelial cells in the presence of inflammatory stimuli is currently still an area of active research [20,22,23]. Understanding the versatile effects of nicotine on epithelial cells and their modification by inflammatory stimuli is important to understand the complex impact of this substance on oral health.

According to the hypothesis that epithelial cell dysfunction may affect the gingival tissue and may facilitate the progression of periodontal disease [19], this study was designed to evaluate the influence of nicotine, *P. gingivalis* lipopolysaccharide (LPS), and tumor necrosis factor (TNF)-α) on epithelial cell proliferation and the expression of proteins involved in the defense function of the epithelium.

## 2. Materials and Methods

### 2.1. Cell Culture

HSC-2 (Oral squamous cell carcinoma cell line, Japan Health Sciences Foundation, Health Science Research Resources Bank, JCRBD 6222) cells were used as a model to investigate the effect of nicotine on epithelial cells [24]. The HSC-2 cultures were maintained in minimal essential medium (MEM, Gibco, Austria) supplemented with 10% fetal bovine serum (FBS, Gibco, Austria) and 1% penicillin/streptomycin (P/S, Gibco, Austria). Cells were cultured in culture flasks at 37 °C in a humidified atmosphere of 5% CO_2_ and 95% air; the culture medium was changed every two days. All experiments were performed using cells between the fifth and eighth passage.

Commercially available standard *P. gingivalis* LPS (InvivoGen, San Diego, CA, USA) was used in the present study. As reported by the supplier and in our recent study [25], this compound contains some lipoprotein traces, which might affect the cell response.

### 2.2. Cell Proliferation/Viability

Cell proliferation/viability was measured by MTT assay similar to a previously described method [26,27]. For each experiment, 2 × 10^4^ cells were seeded to each well of 24-well tissue culture plates in 0.5 mL of MEM supplemented with 10% FBS and 1% P/S. After 24 h, cells were rinsed once with PBS and stimulated with different concentrations of nicotine (10^−8^~10^−2^ M) for another 24 h. Stimulation was performed in MEM supplemented with 1% FBS and 1% P/S. In some experiments, stimulation was done in the presence of *P. gingivalis* LPS in combination with human recombinant soluble CD14 (sCD14, Sigma, St. Louis, MO, USA, 250 ng/mL) [28] or human recombinant TNF-α (PeproTech, Rocky Hill, CT, USA, 10 ng/mL). Cells treated only with MEM supplemented with 1% FBS and 1% P/S served as a control. After incubation for 24 h, 100 µL of 3-(4,5-dimethylthiazol-2-yl)-2,5-diphenyl-tetrazolium bromide (5 mg/mL in PBS, MTT reagent, Sigma, St-Louis, MO, USA) was added to each well, and culture plates were incubated at 37 °C for 4 h. Afterward, the medium was removed, and 500 μL dimethylsulfoxide (DMSO) was added to each well to dissolve formed formazan crystals. The optical density (OD) of the resulting solution was measured at 570 nm using an ELISA Reader (SpectraMax Plus 384, Molecular Devices, San Jose, CA, USA).

### 2.3. Gene and Protein Expression Analyses

HSCs were seeded in 24-well tissue culture plates at a density of 5 × 10^4^ cells per well in 0.5 mL of MEM with 10% FBS and 1% P/S. After 24 h, the culture medium was replaced by MEM supplemented with 1% FBS and 1% P/S and containing different concentrations of nicotine (10^−7^~10^−3^ M) and stimulated for an additional 24 h. In some cases, stimulation was performed in the presence of *P. gingivalis* LPS (1 µg/mL) in combination with sCD14 (250 ng/mL) or TNF-α (10 ng/mL). Non-treated cells were used as the negative control. After stimulation, the gene expression levels of ICAM-1, IL-8, and β-defensin were analyzed by qPCR. Further, the cell surface protein expression of ICAM-1 was analyzed by flow cytometry, and the content of IL-8 protein in the conditioned media was determined by ELISA.

Isolation of total cellular mRNA, its transcription into cDNA, and qPCR were performed using the TaqMan^®^ Gene Expression Cells-to-CT™ kit (Ambion/Applied Biosystems, Foster City, CA, USA) as previously described [29,30]. Real-time PCR was performed on an Applied Biosystems Step One Plus real-time PCR instrument using TaqMan^®^ gene expression assays (all from Applied Biosystems, Foster City, CA, USA) with the following ID numbers: ICAM-1: Hs00164932_m1, IL-8: Hs00174103_m1, β-defensin: Hs00823638_m1, GAPDH: Hs99999905_m1. Triplicate qPCR reactions were prepared for each sample, and the point at which the PCR product was first detected above a fixed threshold (cycle threshold (Ct)) was recorded. The alterations in the gene expression compared to the negative controls were calculated using the 2^−ΔΔCT^ method, taking GAPDH as the housekeeping gene [31].

Analysis of the cell surface protein levels of ICAM-1 in HSCs was performed using a flow cytometer (FACSCalibur, BD Bioscience, CA, USA) [32]. Cells were stained with phycoerythrin-conjugated mouse anti-human ICAM-1 antibody (eBioscience, San Diego, CA, USA; Cat. Nr. 12-0549) or isotype control antibody (Cat. Nr. 12-4714). Cell counting was limited by 10,000 events.

Commercially available Ready-Set-Go! ELISA kits (eBioscience, Waltham, MA, USA) were used to determine protein levels of IL-8 levels in the conditioned medium.

### 2.4. Statistical Analysis

All experiments were repeated at least four times. Data are presented as mean ± SEM. The normal distribution of data was confirmed by the Kolmogorov-Smirnov test (*p* > 0.05 was considered to be normally distributed). One-way analysis of variance (ANOVA) for repeated measures followed by an LSD post hoc test was performed to assess the significance. Values of *p* < 0.05 were considered statistically significant. All data analysis was performed using specific software (SPSS 23.0, SPSS Inc., Chicago, IL, USA).

## 3. Results

### 3.1. Effect of Nicotine on the Proliferation/Viability of HSCs

**Figure 1:**
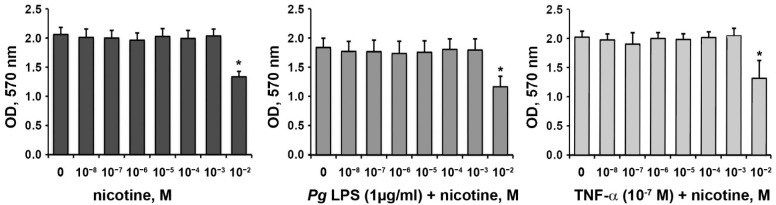
Effect of nicotine on the proliferation/viability of oral epithelial cells. HSC-2 cells were stimulated with different concentrations of nicotine in the presence or in the absence of *P. gingivalis* LPS (*Pg* LPS, 1 µg/mL) or tumor necrosis factor (TNF)-α (10^−7^ M) for 24 h, and the proliferation/viability was measured using MTT assay. The *y*-axis shows optical densities (OD) values measured at 570 nm in five independent experiments. *—significantly different compared to the control.

The effect of nicotine at concentrations of 10^−8^~10^−2^ M on the proliferation/viability of HSCs in the presence or absence of *P. gingivalis* LPS (1 µg/mL) or TNF-α (10^−7^ M), as measured by MTT assay after 24 h of stimulation, is shown in Figure 1. Neither *P. gingivalis* LPS nor TNF-α had any significant effect on HSC-2 proliferation/viability. Under all conditions, nicotine at 10^−2^ M significantly inhibited the proliferation/viability of HSC-2 after 24 h of stimulation. It was reported that salivary nicotine concentrations of tobacco smokers range from 4 × 10^−6^ M to 10^−5^ M [33,34], with much higher levels (0.43–9.62 × 10^−3^ M) being reported in smokeless tobacco users [35]. Concentrations of 10^−8^~10^−2^ M were initially selected in our study to cover possible situations; the 10^−2^ M group was removed because of its de-attaching effect on HSC-2 cells. No significant effects of nicotine at concentrations of 10^−8^~10^−3^ M on HSC-2 proliferation/viability were observed (*p* > 0.05).

### 3.2. Effect of Nicotine on the Expression of IL-8

**Figure 2:**
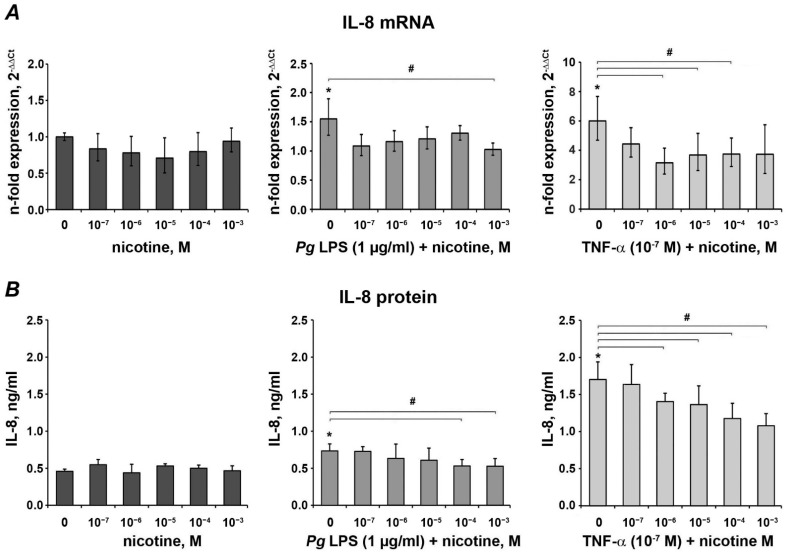
Effect of nicotine on the expression of IL-8 in oral epithelial cells. HSC-2 cells were stimulated with different concentrations of nicotine in the presence or in the absence of *P. gingivalis* LPS (1 µg/mL) or TNF-α (10^−7^ M) for 24 h, and the gene expression (**A**) and the protein content of IL-8 (**B**) in conditioned media were determined by qPCR and ELISA, respectively. Changes in the gene expression levels were calculated using the 2^−ΔΔCT^ method taking GAPDH as an endogenous control gene and non-stimulated cells as a control. The content of IL-8 in the conditioned media after stimulation (**B**) was measured by commercially available ELISA. Each value represents the mean ± S.E.M. of four independent assays. *—significantly different compared to the control. #—significantly different compared to the group without nicotine.

Figure 2 shows the effect of nicotine on IL-8 levels after stimulation for 24 h. In the absence of inflammatory stimuli, nicotine at concentrations of 10^−7^~10^−3^ M had no significant impact on the gene expression levels and protein production of IL-8 after 24 h stimulation. *P. gingivalis* LPS and TNF-α induced a significant increase in the IL-8 gene expression and protein production. Inflammation-induced IL-8 production was generally diminished by all tested concentrations of nicotine. For the *P. gingivalis* LPS-induced response, a significant reduction in IL-8 expression was observed for 10^−3^ M nicotine at the gene level, and 10^−4^~10^−3^ M nicotine at the protein level. For the TNF-α-induced response, significant inhibition was observed starting from 10^−6^ M of nicotine.

### 3.3. Effect of Nicotine on the Expression of ICAM-1

**Figure 3:**
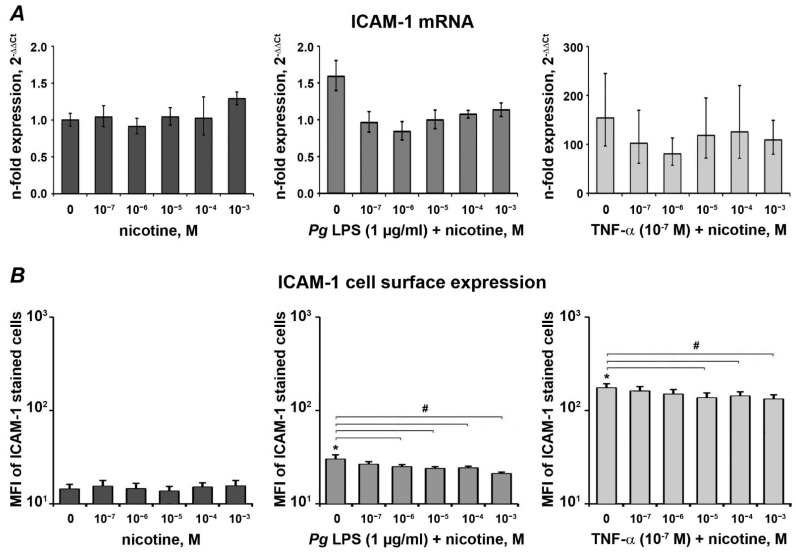
Effect of *P. gingivalis* LPS on the ICAM-1 by oral epithelial cells. HSC-2 cells were stimulated with different concentrations of nicotine in the presence or in the absence of *P. gingivalis* LPS (1 µg/mL) or TNF-α (10^−7^ M) for 24 h, and the gene expression of ICAM-1 (**A**) and the protein content in conditioned media (**B**) were determined by qPCR and flow cytometry, respectively. Changes in the gene expression levels were calculated using the 2^−ΔΔCT^ method taking GAPDH as an endogenous control gene and non-stimulated cells as a control. The amount of ICAM-1 on the cell surface after stimulation (**B**) was measured by flow cytometry based on mean fluorescence intensity (MFI) of ICAM-1 stained cells. Each value represents the mean ± S.E.M. of four independent assays. *—significantly different compared to the control. #—significantly different compared to the group without nicotine.

The effect of nicotine on the basal, *P. gingivalis* LPS, and TNF-α-induced ICAM-1 expression in HSC-2 cells is displayed in Figure 3. Neither gene expression (Figure 3A) nor cell surface expression was affected by nicotine at all tested concentrations in the absence of inflammatory stimuli. Both *P. gingivalis* LPS and TNF-α induced a significant increase in ICAM-1 expression at both gene and protein levels. Nicotine diminished *P. gingivalis* LPS and TNF-α induced gene expression, but this effect was not statistically significant. The cell surface expression of ICAM-1 induced by *P. gingivalis* LPS and TNF-α was decreased by nicotine in a concentration-dependent manner. Significant differences were observed for 10^−6^~10^−3^ M nicotine in the presence of *P. gingivalis* LPS and 10^−5^~10^−3^ M in the presence of TNF-α.

### 3.4. Effect of Nicotine on the Expression of β-Defensin

**Figure 4:**
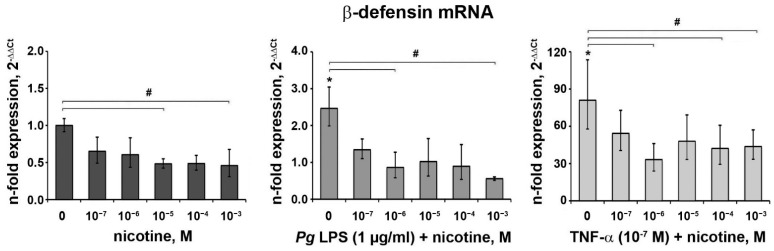
Effect of nicotine on the expression of β-defensin in oral epithelial cells. HSC-2 cells were stimulated with different concentrations of nicotine in the presence or in the absence of *P. gingivalis* LPS (1 µg/mL) or TNF-α (10^−7^ M) for 24 h. Changes in the gene expression levels were calculated using the 2^−ΔΔCT^ method taking GAPDH as an endogenous control gene and non-stimulated cells as a control Data are presented as the mean ± S.E.M. of three independent experiments. *—significantly different compared to the control. #—significantly different compared to the group without nicotine.

The effect of nicotine on the gene expression of β-defensin with or without *P. gingivalis* LPS or TNF-α is shown in Figure 4. Both *P. gingivalis* LPS and TNF-α significantly increased the gene expression of β-defensin. Nicotine induced an inhibitory effect on β-defensin expression at all tested conditions. The basal β-defensin expression was significantly decreased by 10^−5^ and 10^−3^ M of nicotine. In the presence of *P. gingivalis* LPS, a significant effect of nicotine was observed at the concentrations 10^−6^ and 10^−3^ M. TNF-α-induced β-defensin expression was significantly affected by 10^−6^, 10^−4^, and 10^−3^ M nicotine.

## 4. Discussion

It is widely accepted that smoking is a significant risk factor for periodontitis, and several mechanisms have been proposed to explain this association [36]. Cigarette smoke has been shown to induce autophagy and apoptosis of oral epithelial cells [37], reduce immune defense, delay the healing process, and disrupt collagen synthesis and epithelium structure [38,39]. Among the plentiful research, many studies were conducted solely on the effect of nicotine on cell viability, cell attachment/adhesion, and inflammatory mediator production of different oral cells such as periodontal ligament cells, epithelial cells, and endothelial cells [19]. Predominantly, nicotine posed a negative effect on the aforementioned aspects. However, the oral environment contains numerous bacteria and host-derived inflammatory mediators, and therefore it is clinically relevant to investigate the effect of nicotine in the presence of different stimuli. Furthermore, the impact of nicotine on oral tissue might be modified by environmental factors.

We did not observe any significant effect of nicotine at concentrations up to 10^−3^ M on the proliferation/viability of HSC-2. This is consistent with previous studies reporting no statistically significant effects of nicotine at a concentration of 10^−7^ to 10^−3^ on gingival epithelial cells [40] and 10^−6^ M on gingival fibroblasts [41]. However, nicotine at a higher concentration (3.7 × 10^−3^ M) inhibited gingival fibroblasts proliferation/viability [42], which is also in line with our data. Thus, it seems that nicotine at concentrations higher than 10^−3^ M has a strong toxic effect on the different cell types. Therefore, we excluded the group with 10^−2^ nicotine from further analysis.

In our study, we did not observe any effect of *P. gingivalis* LPS and TNF-α on the proliferation/viability of HSC-2 cells. This observation is not always in agreement with the previous reports. Thus, *P. gingivalis* LPS, at concentrations similar to that used in our study, enhanced the proliferation/viability of primary human oral epithelial cells [43], but did not affect that of oral squamous carcinoma cells [44]. TNF-α at a concentration of 100 ng/mL was shown to induce the apoptosis of primary gingival epithelial cells [45]. It seems that the effect of the different inflammatory stimuli on epithelial cell viability depends on the cell source, and this fact should be considered by the data interpretation.

IL-8 is crucial in the local defense against bacteria because it functions as a chemokine directing neutrophil migration [8]. The modulation of IL-8 secretion in epithelial tissues during episodes of periodontitis is considered a key component for the maintenance of oral health [46]. IL-8 secretion following nicotine treatment at 10^−7^ to 10^−3^ M was not significantly different compared with the control group, which is consistent with results obtained in primary gingival keratinocytes [22]. However, other studies show that nicotine increased IL-8 production in cultured human oral epidermoid carcinoma cell lines (10^−6^ to 10^−4^ M) [47] and gingival epithelial cells (3 × 10^−4^ to 10^−3^ M) [48]. Differences among our data and other mentioned studies may be related to cell variability and different experimental conditions.

Similar to IL-8, we did not observe any effect of nicotine on the gene and surface expression of ICAM-1. ICAM-1 is an adhesion molecule, which is involved in the transepithelial migration of leukocytes into the gingival sulcus [49]. There is no study on the effect of nicotine on ICAM expression in oral epithelial cells, to the best of our knowledge. Our previous study showed that nicotine had no significant effect on ICAM-1 expression in human umbilical vein endothelial cells [27], which is in agreement with the current observation.

In contrast to basal expression, nicotine diminished *P. gingivalis* LPS or TNF-α induced expression of IL-8 and ICAM-1. At afirst look, this observation is somewhat surprising. There are several reports that inflammation is generally aggravated by the combined effect of smoking and bacterial infection [27,50,51,52,53], which was echoed by two aspects. First, there is clinical evidence that tobacco users with poor oral hygiene have more severe periodontal destruction [54]. Second, the activation of nicotine receptors might upregulate the gene expression of NF-κB [55]. However, the antagonistic effect of nicotine on inflammation was also shown in several studies [56,57,58,59]. The mechanisms underlying this phenomenon are the activation of α7-nAChR (α7-nicotinic acetylcholine receptor) [60] and suppression of the Th1 and Th17 response [61]. Hypersensitivity to LPS and excessive production of IL-1 and IL-6 were observed in α7 nAChR-deficient mice [62]. Activation of α7-nAChR in monocytes can inhibit the degradation of the inhibitory IkB protein and prevent NF-κB translocation and activation [63]. Clinically, nicotine exposure probably reduced the pro-inflammatory cytokine burden, which promoted bacterial survival and invasion and further aggravation of the periodontitis [64]. Well-recognized harmful effects of smoking might outweigh some beneficial aspect brought about by nicotine. As for our study, HSC-2 cells might be responsible for the result; however, an up-to-date search only returned one paper revealing that nicotine promoted HSC-2 cell growth and migration through epidermal growth factor signaling [65].

β-defensins, categorized as antimicrobial peptides against bacterial, viral, and fungal infections, are mainly produced by epithelial cells when stimulated with infectious stimuli [10,66]. The expression, location, and function of β-defensins have been extensively investigated in oral science. β-defensin 1 gene polymorphism was associated with dental caries in permanent dentition, according to a meta-analysis [67]. Noteworthy, β-defensins could be viewed as major players in maintaining epithelial function and homeostasis. Increased secretion of β-defensins was shown in human mucosal epithelial cells under the exposure of whole cigarette smoke [68]; a decreased level was reported at the induction phase of an experimental gingivitis model [69]. Additionally, the β-defensin 2 level in the gingival tissue and gingival crevicular fluid was higher in the chronic periodontitis group than in the gingivitis group [70] and healthy group [71]. Furthermore, β-defensins contribute to the healing process of wounds by promoting keratinocyte migration and proliferation [72] and act as a chemoattractant, guiding blood cells to the site of infection [73]. Few papers focused on the effect of nicotine on β-defensin expression [48,74,75]. Exposure to nicotine (3 × 10^−4^ to 10^−3^ M) caused substantial inhibition of TNF-α, and *P. gingivalis* LPS stimulated β-defensin 2 production in human gingival epithelial cells [48,74], which, to some extent, is congruent with our findings, regardless of the different stimulant dosages (*P. gingivalis* LPS, 50 µg/mL; TNF-α, 30 ng/mL) and cell type (primary gingival epithelial cells; HaCaT keratinocytes). Our results strongly suggest that nicotine reduces antimicrobial activity and may allow overgrowth and invasion of potential periodontal pathogens.

The study provided us the probability that nicotine might serve as an anti-inflammatory agent in certain cell types such as HSC-2 cells; however, the suppressive effect on β-defensin expression should not be neglected. Different signaling pathways may be involved in these two distinct observations. It should be noted that in our study, we used the oral squamous carcinoma cell line as a model of oral epithelium. This was done mainly because this line exhibits stable properties during culturing. However, a potential anti-inflammatory effect of nicotine on oral epithelium should be confirmed using primary and/or immortalized cells. In our study, the cells were exposed to nicotine only briefly, which is not physiologically relevant. Further research on the mechanism and effect of chronic exposure to nicotine is still warranted.

## 5. Conclusions

HSC-2 cell viability was not impaired by nicotine at the concentrations usually observed in smokers; increased expressions of IL-8 and ICAM-1 induced by *P. gingivalis* LPS or TNF-α were diminished by nicotine treatment. Additionally, an inhibitory effect on β-defensin production was also demonstrated. Apart from being the usually alleged harmful substance, nicotine probably exerted a suppressive effect on inflammatory factors production in HSC-2 cells.

## Data Availability

The data presented in this study are available on request from the corresponding author.
